# Real-time detection of DNA topological changes with a fluorescently labeled cruciform

**DOI:** 10.1093/nar/gkt413

**Published:** 2013-05-16

**Authors:** Kevin M. Jude, Abbey Hartland, James M. Berger

**Affiliations:** Department of Molecular and Cellular Biology, California Institute for Quantitative Biosciences, University of California, Berkeley, CA 94720-3220, USA

## Abstract

Topoisomerases are essential cellular enzymes that maintain the appropriate topological status of DNA and are the targets of several antibiotic and chemotherapeutic agents. High-throughput (HT) analysis is desirable to identify new topoisomerase inhibitors, but standard *in vitro* assays for DNA topology, such as gel electrophoresis, are time-consuming and are not amenable to HT analysis. We have exploited the observation that closed-circular DNA containing an inverted repeat can release the free energy stored in negatively supercoiled DNA by extruding the repeat as a cruciform. We inserted an inverted repeat containing a fluorophore-quencher pair into a plasmid to enable real-time monitoring of plasmid supercoiling by a bacterial topoisomerase, *Escherichia coli* gyrase. This substrate produces a fluorescent signal caused by the extrusion of the cruciform and separation of the labels as gyrase progressively underwinds the DNA. Subsequent relaxation by a eukaryotic topoisomerase, human topo IIα, causes reintegration of the cruciform and quenching of fluorescence. We used this approach to develop a HT screen for inhibitors of gyrase supercoiling. This work demonstrates that fluorescently labeled cruciforms are useful as general real-time indicators of changes in DNA topology that can be used to monitor the activity of DNA-dependent motor proteins.

## INTRODUCTION

Emerging resistance to available antibacterial agents, along with the undesirable side effects of many existing antitumor agents, underscore an urgent need for therapeutic compounds that have novel chemical properties (1,2). Success in developing new compounds is expected to be facilitated by the availability of proven drug targets and robust high-throughput (HT) screening methods (3). DNA topoisomerases have proven to be a particularly useful family of targets for small-molecule inhibitors (4–6). Among these inhibitors are the fluoroquinolones (7,8), which are leading antibacterial agents, and the popular anticancer compounds camptothecin, doxyrubicin and etoposide (9–11,5).

Topoisomerases are divided by their mechanism of action into two classes, type I and type II, and categorized further by specific subtypes (12,13). Type II topoisomerases use the energy of ATP hydrolysis to drive DNA cleavage and strand passage that allow a variety of activities such as introduction or removal of supercoils, removal of knots and disentangling of catenated DNA. Gyrase, a bacterial type II topoisomerase, has the unique ability to introduce negative supercoils into DNA (14). Gyrase is a proven drug target that can either be converted to a poison by small molecules (e.g. fluoroquinolones) that stabilize the DNA cleavage state, or be catalytically inhibited by other small molecules (e.g. aminocoumarins) that inhibit the ATPase reaction and block strand passage (15,16). Both poisons and catalytic inhibitors block the introduction of supercoils (16–18), which makes inhibition of supercoiling the most general assay for antigyrase agents. Notably, limited cross-reactivity exists between many types of inhibitors of prokaryotic and eukaryotic type II topoisomerases, and inhibitors of human and bacterial topoisomerases have become successful disease-specific therapeutic agents. For example, bacterial topoisomerase inhibitors (fluoroquinolones) are among the most prescribed antimicrobials in the USA, while human topo II inhibitors, such as doxorubicin and etoposide, are commonly prescribed antitumor agents. Unfortunately, resistance is eroding the utility of quinolone-type compounds (19), whereas the antitumor agents exhibit general toxicity as well as therapeutic benefit (20). Thus, there is an imperative to develop new classes of type II topoisomerase inhibitors (21,22).

**Figure 1.**
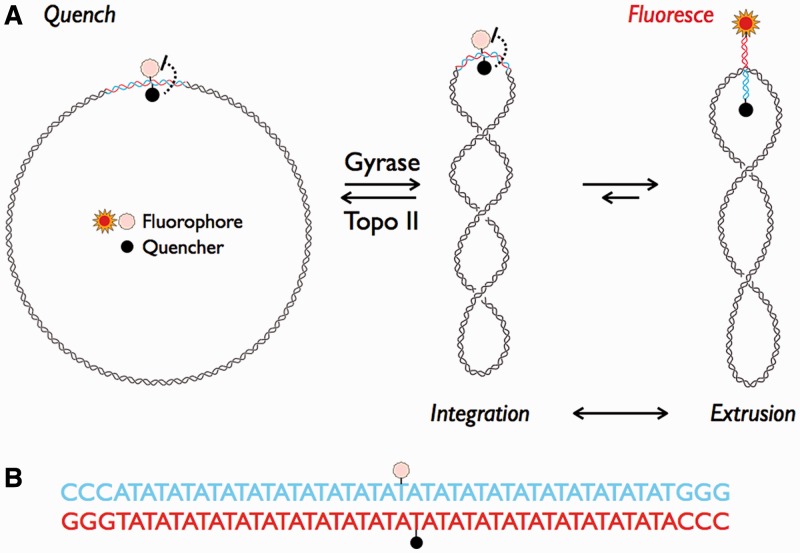
(**A**) Schematic representation of cruciform extrusion due to negative supercoiling. Plasmid pAT42C contains a 42-bp AT repeat (red and blue) labeled on opposing strands with a fluorophore (fluorescein) and quencher (dabsyl). Treatment with gyrase introduces negative supercoiling, which extrudes the repeat as a cruciform. The separation of fluorescein and dabsyl by cruciform formation allows detection of a fluorescent signal. Topo II can remove supercoils, causing an extruded and fluorescent cruciform to reintegrate. (**B**) The sequence of the inserted repeat; the sites of fluorophore and quencher modification are indicated by stars as in (A).

In a standard gyrase supercoiling assay, relaxed and supercoiled DNA species produced by the enzyme are resolved on agarose gels. Gel electrophoresis is both time-consuming and labor intensive, making it unsuitable for large-scale inhibition studies. HT assays for supercoiling do exist but rely on indirect reporters [e.g. ethidium bromide intercalation (23), or DNA triplex formation (24)]. The ethidium bromide intercalation assay suffers from a low signal-to-noise ratio, while the triplex formation assay undergoes a drift in signal that has been attributed to either slow binding of the oligonucleotide to the supercoiled plasmid or damage to the supercoiled product; both assays are end point assays and require quenching of the reaction before readout. To overcome these bottlenecks, we developed a robust HT assay for DNA supercoiling that is suitable for the discovery of new classes of topoisomerase inhibitors. Our assay takes advantage of the fact that DNA cruciform extrusion and reintegration accompany changes in DNA supercoiling (25,26). In the substrate reported here, cruciform extrusion results in separation of a fluorophore and quencher, allowing detection of a fluorescent signal produced by a negatively supercoiled plasmid (Figure 1A). We show that this reaction generates a stable product with excellent resolution of relaxed and supercoiled species that can be monitored in a high-density format in real time.

## MATERIALS AND METHODS

### Preparation of the cruciform-forming plasmid

Plasmid pUC19AB was prepared by introducing two point mutations into pUC19 by QuickChange mutagenesis (Agilent, Santa Clara, CA) using the primers GAATTCGGGCTCGGTACTCGGGGATCCTCTAGAG, CTCTAGAGGATCCCCGAGTACCGAGCCCGAATTC, CCAGTGAATTCGGGCTCGGTACCCG and CGGGTACCGAGCCCGAATTCACTGG (Integrated DNA Technologies, San Diego, CA). The nucleotide sequence was confirmed by UC Berkeley DNA Sequencing Facility. pUC19AB was prepared using a Nucleobond PC 10000 kit (Macherey-Nagel, Bethleham, PA) and linearized by digestion with BanII and AvaI (New England Biolabs, Ipswich, MA; 1.6 units/µg DNA) for 6 h at 37°C. The linearized DNA was repurified by chromatography on a Poros HQ column in 0.5 M NaCl, 50 mM Tris, pH 7.9, and 5 mM EDTA and eluted with 1 M NaCl.

5′-phosphorylated cruciform-forming oligonucleotides 5′-CCGACAGCACGAGCCCATATATATATATATATATATA[Dabcyl-dT]ATATATATATATATATATATGGGCCAACCAACCAGCC-3′ and 5′-GGTTGGTTGGCCCATATATATATATATATATA[Fluorescein-dT]ATATATATATATATATATATATGGGCTCGTGCTG-3′ (Midland Certified Reagent Company, Midland, TX) were purified by polyacrylamide gel electrophoresis, lyophilized and dissolved in 10 mM Tris, pH 7.5, 5 mM MgCl_2_, 1 mM EDTA. The oligonucleotides were annealed by slow cooling from 95 to 16°C over 80 min in a thermocycler (Eppendorf, Hauppauge, NY). Plasmid pAT42C was prepared by ligation of the annealed oligonucleotides into linearized pUC19AB. pUC19AB (7.5 ng/µl) was mixed with equimolar annealed oligonucleotides in 50 mM Tris, pH 7.5, 10 mM MgCl_2_, 10 mM dithiothreitol (DTT) and 1 mM ATP and ligated by incubation with T4 DNA ligase (New England Biolabs; 62.5 U/µg DNA) for 40 h at room temperature. The ligation reaction was stopped by heating to 65°C for 20 min and then was concentrated by diafiltration using Centricon Plus-70 centrifugal filter units (EMD Millipore, Billerica, MA). Circularized relaxed pAT42C was purified by preparative agarose gel electrophoresis in a Prep Cell (Bio-Rad, Hercules, CA) using 1.2% agarose in Tris-acetate-EDTA buffer containing 1 µg/ml chloroquine. Fractions containing circularized DNA were concentrated by diafiltration. Typical yields of circularized plasmid after purification were 10–15%.

### Preparation of topoisomer distributions for 2D gels

To prepare topoisomer distributions of pUC19AB and pAT42C, the plasmids were nicked with Nt.BspQI (New England Biolabs). The plasmids were religated for 75 min at room temperature in the presence of 0–9 µg/ml ethidium bromide. Ethidium bromide was removed by phenol:chloroform extraction, followed by ethanol precipitation in the presence of ammonium acetate. The supercoiling density at which cruciform extrusion occurs was calculated as 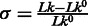, where *Lk* is the linking number at which a discontinuity was observed in the 2D gel and *Lk^0^* = 264 for the 2747 base pair pAT42C plasmid.

### Protein purification

*Escherichia coli* GyrA and GyrB were purified separately as described (27). Briefly, the histidine-tagged proteins were expressed in *E.coli* BL21-CodonPlus(DE3)-RIL cells (Agilent). After initial purification using a Ni-NTA column (GE Life Sciences, Pittsburg, PA), the histidine tags were cleaved by digestion with tobacco etch virus protease, and the proteins were repurified on a Ni-NTA column. After size exclusion chromatography using an S200 column (GE Life Sciences), the proteins in 500 mM KCl, 30% (v/v) glycerol, 1 mM DTT, 1 mM EDTA, 50 mM Tris, pH 7.5, were flash frozen in liquid N_2_.

Human topo IIα was expressed in BCY123 yeast cells transformed with pCM1 FlhTopoIIα (28). Cells were lysed by mechanical grinding under liquid N_2_, and lysates were then resuspended in buffer L (25 mM KCl, 50 mM Tris, pH 7.7, 10% (v/v) glycerol, 1 mM EDTA, 1 mM EGTA, 1 mM 2-mercaptoethanol, 1 mM phenylmethanesulfonyl fluoride, 1 µg/ml leupeptin and 1 µM pepstatin A). Resuspension and subsequent purification steps were performed at 4°C. The lysate was centrifuged for 30 min at 25 000*g*. Polyethyleneimine pH 7.5 was added dropwise to the supernatant from a 10% (w/v) stock to 0.2% final concentration while stirring. The suspension was centrifuged for 10 min at 12 000*g*, the pellet was resuspended in buffer L1000 (L + 1 M KCl), and the lysate was centrifuged again.

While stirring, ammonium sulfate was added to the supernatant to 35% (w/v) final concentration. The suspension was centrifuged for 25 min at 25 000*g*, and additional ammonium sulfate was added to the supernatant to 65% (w/v) final concentration. The suspension was centrifuged again, and the pellet was dissolved in buffer L. The solution was injected onto a Poros HS column (Life Technologies, Grand Island, NY) and eluted with a gradient to 100% buffer L1000. Peaks containing topo IIα were identified by sodium dodecyl sulphate–polyacrylamide gel electrophoresis, concentrated and dialyzed overnight against buffer L.

After dialysis, sufficient buffer L1000 was added to the protein to increase [KCl] to 125 mM. The protein was purified using a HiTrap Q HP column (GE Life Sciences) and eluted with a gradient to 100% buffer L1000. Fractions containing topo IIα were concentrated and purified using a Sephacryl S300 column (GE Life Sciences) equilibrated with buffer GFT2 (500 mM potassium acetate, 50 mM Tris, pH 7.9, 1 mM EDTA, 1 mM EGTA, 10% (v/v) glycerol, 1 mM DTT). Pure human topo IIα was concentrated to 34 µM and then diluted with an equal volume of storage buffer (GFT2 + 70% (v/v) glycerol). Aliquots were flash frozen in liquid nitrogen.

### Preparation of supercoiled pAT42C

Supercoils were introduced into pAT42C (10 µg/ml) at 37°C in supercoiling buffer (10 mM Tris, pH 7.9, 5 mM HEPES, pH 7.5, 70 mM KCl, 0.1 mg/ml bovine serum albumin, 6 mM MgCl_2_, 0.6 mM DTT, 13% (v/v) glycerol, 1 mM ATP) for 30 min in the presence of 3 nM *E.coli* gyrase holoenzyme. The reaction was stopped by the addition of SDS to 1% (w/v) and EDTA to 25 mM. Precipitated potassium dodecyl sulfate was removed by centrifugation, and the supercoiled plasmid was purified using a NucleoSpin Extract II kit (Macherey-Nagel).

### HT assay replicate-experiment study

All HT liquid handling operations were performed using a Biomek 3000 liquid handling robot (Beckman Coulter, Brea, CA). Inhibitors listed in Table 1 were purchased from Sigma Aldrich (St. Louis, MO) except for gemifloxacin and moxifloxacin (OChem, Des Plains, IL), and serial dilutions were prepared as 4× stock concentrations in 4% (v/v) dimethyl sulfoxide (DMSO). Supercoiling reactions were performed in Corning 3820 384-well microplates (Corning, Tewksbury, MA) in buffer HT (supercoiling buffer with 5% (v/v) glycerol, 0.1% (v/v) Tween-20) with inhibitors at 1× concentration or 1% DMSO, 0.92 ng/µl relaxed pAT42C and 0.6 nM gyrase. Master mix (16 µl/well) containing enzyme and substrate was dispensed to the microplates, followed by 4× stocks of inhibitors or DMSO (8 µl/well). Reactions were started by addition of ATP to 1 mM (8 µl/well) in 32 µl and were incubated at 37° for 30 min before the plates were read in a Victor 3 V plate reader (Perkin Elmer, Waltham, MA). Water (8 µl) was added to negative control reactions in the place of ATP. Fluorescence for each titration series was normalized to the range of the positive (no inhibitor) and negative (no ATP) control reactions. This experiment was repeated after on 2 separate days. To determine the potency of each inhibitor, inhibition isotherms were fit to the equation 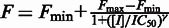, where *F* is normalized fluorescence, *F*_max_ = 1, *F*_min_ = 0, *[I]* is the inhibitor concentration and *γ* is a slope parameter. For each inhibitor, the difference in log potencies measured on 2 days was calculated as 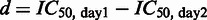 with mean difference 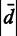 and standard deviation *s_d_.* The mean ratio (MR) for the two-day replicate experiment was calculated as 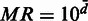. The smallest statistically significant potency ratio between two compounds was calculated as 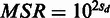. Limits of Agreement were calculated as 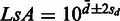. For each compound, the geometric mean potency ratio (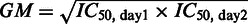) was calculated. The Z’ factor for the assay was calculated as 
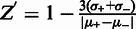
, where µ_+_ and µ_−_ are the mean values of the positive and negative controls, and σ_+_ and σ_−_ are the standard deviations of the positive and negative controls. In a typical trial of the replicate-experiment study, µ_+_ = 2588, σ_+_ = 119, µ_−_ = 10 391, and σ_−_ = 356

**Table 1.** IC_50_s determined in replicate cruciform extrusion experiments and in gel-based assay

Inhibitor	Abbreviation	IC_50, day 1_ (µM)	IC_50, day 2_ (µM)	IC_50, gel_ (µM)
Pipemidic acid	Pipe	80	74	n.d.
Nalidixic acid	Nal	210	200	n.d.
Oxolinic acid	Oxo	142	80	n.d.
Enoxacin	Enox	5.6	4.8	n.d.
Norfloxacin	Nor	3.3	2.9	n.d.
Ciprofloxacin	Cipro	1.4	1.3	1.2
Levofloxacin	Levo	2.5	2.2	n.d.
Pazufloxacin	Pazu	4.0	3.3	n.d.
Sparfloxacin	Spar	2.0	1.6	n.d.
Trovafloxacin	Trova	1.9	1.8	n.d.
Gemifloxacin	Gemi	1.0	1.1	n.d.
Prulifloxacin	Pruli	1.7	1.4	n.d.
Moxifloxacin	Moxi	2.8	2.4	4.6
Novobiocin	Novo	2.8	2.6	n.d.
Pipemidic acid 2	Pipe2	75	58	n.d.
Enoxacin 2	Enox2	6.1	5.1	n.d.
Norfloxacin 2	Nor2	2.8	2.7	n.d.
Ciprofloxacin 2	Cipro2	1.3	1.2	n.d.
Levofloxacin 2	Levo2	1.7	1.7	n.d.
Pazufloxacin 2	Pazu2	4.2	3.2	n.d.

n.d., not determined

### Screening of the NIH clinical collection

The NIH Clinical Collection (NCC) screen of 482 compounds was purchased from Evotec (South San Francisco, CA), and reagents were diluted to a 4× concentration of 200 µM in 4% DMSO. Reactions were performed as for the replicate-experiment study.

## RESULTS

### Preparation of the cruciform-forming plasmid for detection of supercoiling by fluorescence

Because the hairpins of DNA cruciforms may be separated by the torsional strain of negative supercoils (25,29,30), we reasoned that an inverted repeat of the appropriate sequence, labeled with a fluorophore and a quencher, would produce a fluorescent signal on supercoiling and be quenched on relaxation. The degree of supercoiling required to induce cruciform formation is sequence dependent, so we chose to use a 42 bp AT repeat that has been shown to form a cruciform at a supercoiling density of σ = −0.04 (26) (see ‘Materials and Methods’ section). To prepare a suitable plasmid (pAT42C, Figure 1), we inserted this repeat, labeled at adjacent sites on opposite strands with fluorescein and dabcyl, into the AvaI/BanII site of pUC19AB. To separate closed-circular DNA from nicked and linear forms, we purified pAT42C by gel electrophoresis in the presence of the intercalating agent chloroquine. Removal of chloroquine from the purified DNA was confirmed by measuring UV absorption at 344 nm and by the detection of relaxed DNA species by agarose gel electrophoresis (data not shown).

**Figure 2.**
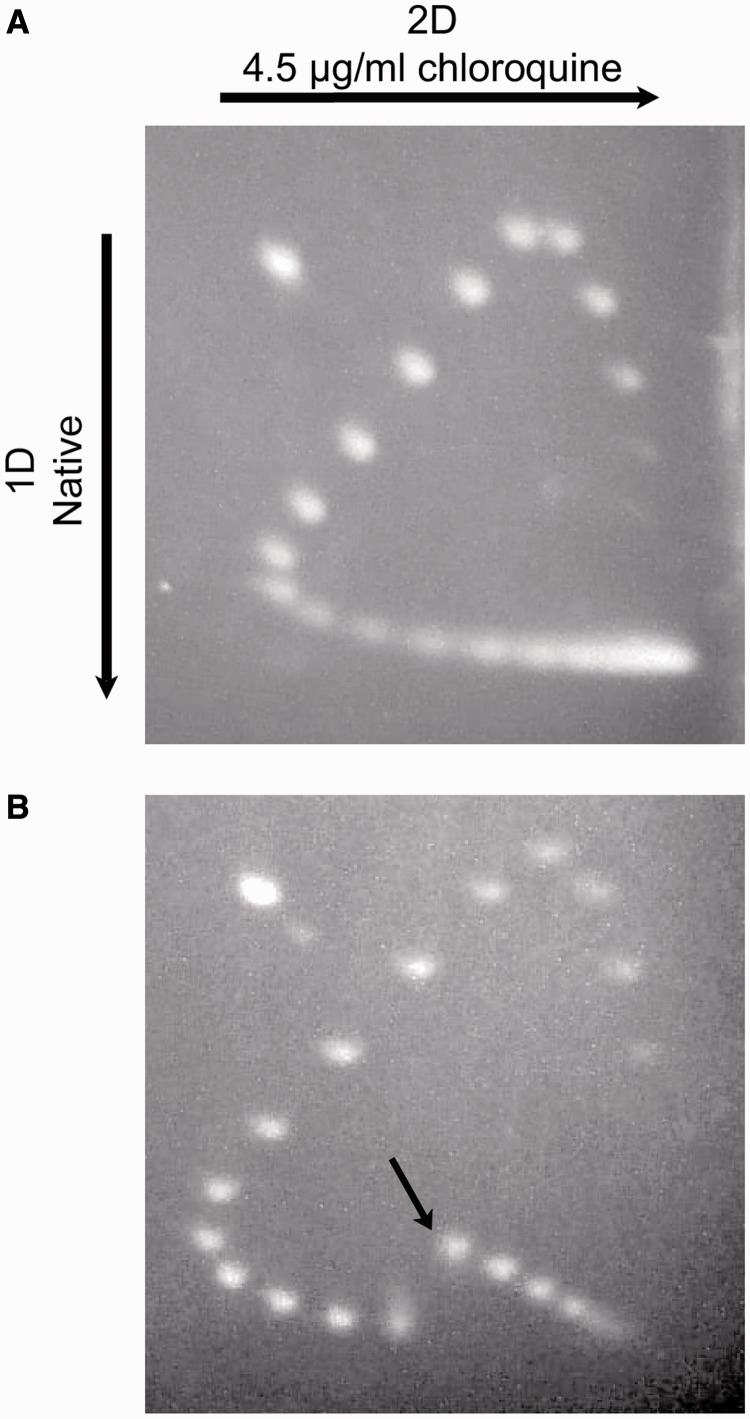
Demonstration of cruciform extrusion by 2D electrophoresis of topoisomers. Topoisomer distributions of pUC19AB (**A**) and pAT42C (**B**) were displayed by electrophoresis in 1.2% agarose gels in tris-phosphate-EDTA (TPE) buffer for 18 h at 70 V. The gels were soaked in several changes of TPE plus 4.5 µg/ml chloroquine, then rotated 90° and subjected to electrophoresis in TPE plus chloroquine for an additional 18 h at 70 V. The gels were stained with ethidium bromide. pAT42C exhibits a discontinuity between ΔLk = 10 and ΔLk = 11, signifying cruciform extrusion (indicated in panel B with an arrow).

The extrusion of cruciforms from negatively-supercoiled DNA is accompanied by an increase in writhe, which decreases plasmid mobility during electrophoresis in agarose gels. To confirm that negative supercoiling causes extrusion of the AT42 cruciform used here, we first prepared a broad distribution of pAT42C topoisomers by nicking the plasmid and then religating it in the presence of increasing concentrations of ethidium bromide. Intercalation of ethidium bromide introduces positive supercoils to DNA in a concentration-dependent manner, and subsequent removal of the intercalator during purification results in negatively supercoiled DNA. Two-dimensional gel electrophoresis of the pAT42C topoisomer distribution revealed a discontinuity in plasmid migration between ΔLk=10 and ΔLk=11, showing that the cruciform is completely integrated at σ > −0.04 and completely extruded at σ < −0.04 (see ‘Materials and Methods’ section) (Figure 2). The discontinuity was not observed with the parental plasmid pUC19AB treated in the same manner. Thus, our starting plasmid substrate responds physically to alterations in supercoiling levels as planned.

**Figure 3.**
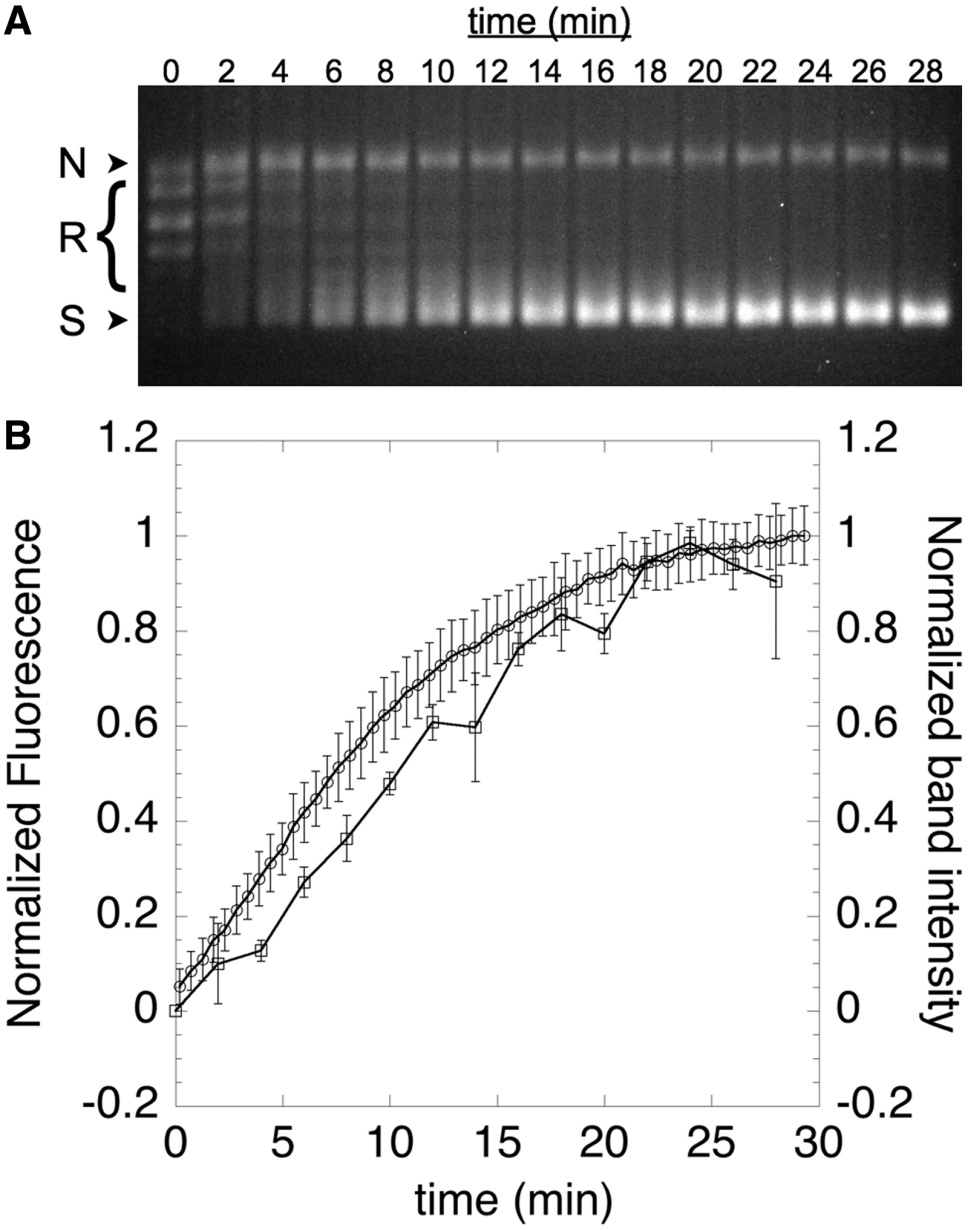
Time course of cruciform extrusion. Negative supercoils were introduced into labeled pAT42C (3.68 ng/µl) by treatment with 0.6 nM gyrase at 30°C; aliquots were removed at the indicated time points and quenched with SDS and EDTA before analysis by agarose gel electrophoresis; nicked, relaxed and supercoiled DNA bands are indicated as N, R and S, respectively (**A**). The appearance of the fully supercoiled species in the gel was quantified and expressed as a function of time (**B**, squares). Fluorescence was also monitored continuously by fluorescence in solution (B, circles). The close correspondence between the introduction of supercoils and the increase in fluorescence validates use of fluorescence as a measure of supercoiling. Error bars represent the standard deviation of three replicates.

**Figure 4.**
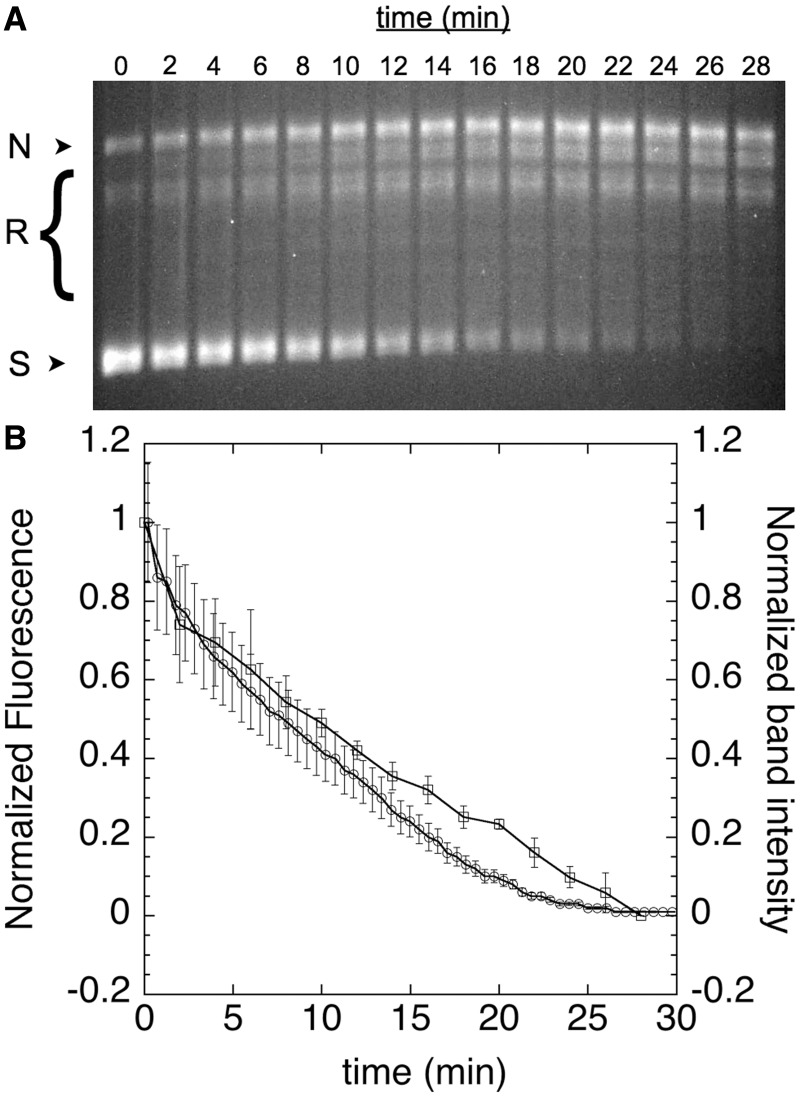
Reintegration of cruciform. Supercoiled labeled pAT42C (3.68 ng/µl) was relaxed with human Topo IIα (1.6 nM) at 37°C; aliquots were removed at the indicated time points and quenched with SDS and EDTA before analysis by agarose gel electrophoresis; nicked, relaxed and supercoiled DNA bands are indicated as N, R and S, respectively (**A**). The disappearance of supercoiled bands in the gel was quantified and expressed as a function of time (**B**, squares). Fluorescence was also monitored continuously in solution (B, circles). Error bars represent the standard deviation of three replicates.

To confirm that the labeled cruciform gives rise to fluorescence in a supercoiling-dependent manner, we next performed two time-course assays that monitored fluorescence and DNA topology in parallel (Figure 3). We used gyrase to generate negatively supercoiled DNA, which was detected by gel electrophoresis and appeared at the same rate as cruciform formation, which we monitored continuously in microtiter plates as an increase in fluorescence (see ‘Materials and Methods’ section). This result demonstrates that supercoiling of pAT42C by gyrase can be observed in real time using our fluorescently labeled cruciform substrate. Similarly, in the reverse reaction, relaxation of supercoiled DNA by human topo IIα, as evidenced by the disappearance of supercoiled species by gel electrophoresis, can be seen to occur simultaneously with the fluorescence quenching of labeled pAT42C in the plate-based assay (Figure 4).

### HT assay replicate-experiment study

**Figure 5.**
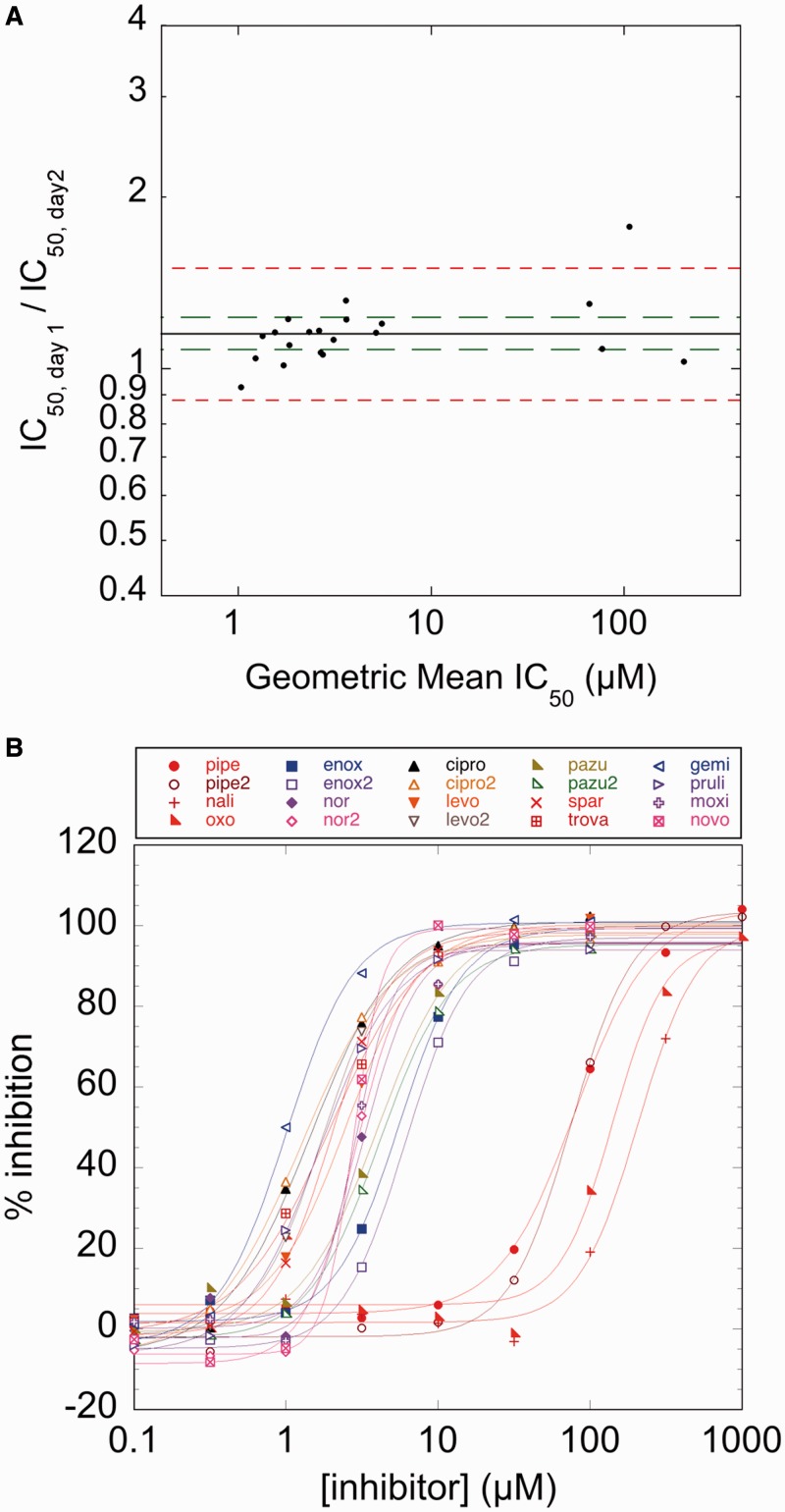
Replicate-experiment study. (**A**) The ratios of IC_50_s measured on two separate days are plotted against the geometric mean of the IC_50_s measured for each compound (blue diamonds). The MR for all compounds is 1.15 (blue line) with a 95% confidence interval of 1.08–1.23 (green dashed lines). Because the MR is close to 1 and the 95% confidence interval is narrow, no statistically significant difference exists between the two experimental trials. The limits of agreement between replicates are 0.88–1.50 (red dashed lines), representing the individual compound variation between two trials. (**B**) Inhibition isotherms from a typical trial of the replicate-experiment study. Percent inhibition was normalized by uninhibited reactions containing 0 and 1 mM ATP as upper and lower limits, respectively. Abbreviations of compounds are defined in Table 1.

**Figure 6.**
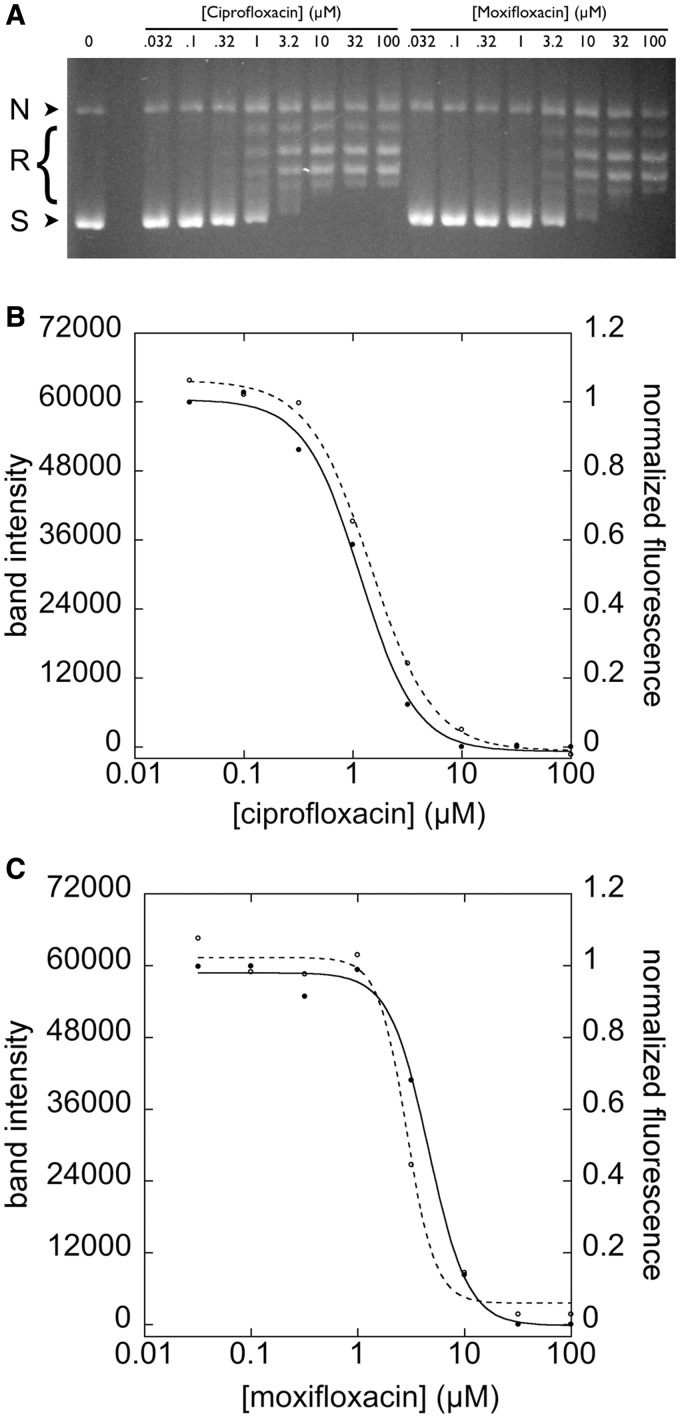
Comparison of drug responses by gel-based and cruciform extrusion assays. Labeled pAT42C (3.68 ng/µl) was supercoiled by incubation with 0.6 nM gyrase in the presence of the indicated concentrations of ciprofloxacin or moxifloxacin. Nicked, relaxed and supercoiled DNA bands are indicated as N, R and S, respectively (**A**). After quenching with SDS and EDTA, the reaction products were analyzed by electrophoresis in a 1% agarose gel. Inhibition isotherms were plotted based on the intensity of the supercoiled band (dashed lines, panels **B** and **C**). Fluorescence-based cruciform extrusion assays were also performed in solution for each fluoroquinolone and are plotted as solid lines in panels B and C.

Having established that a labeled cruciform-forming sequence can be used to assess both supercoiling state and topoisomerase activity, we next set out to test the reproducibility and robustness of the substrate and our assay. To assess the variability of a HT assay and to show reproducibility over a range of potencies, a replicate-experiment study is typically performed. In a replicate-experiment study, IC_50_ measurements of compounds spanning a range of potencies are measured in duplicate and compared to determine both a MR and a minimum significant ratio (MSR). The MR is the geometric average of the ratios between IC_50_s measured for each compound in two trials and provides a measure of reproducibility. The MSR is the smallest ratio of IC_50_s between two compounds that can be statistically significant and thus is a measure of the ability of an assay to rank-order compounds by efficacy (31,32). We first assayed supercoiling of pAT42C by *E.coli* gyrase in 384-well plates by monitoring the increase of fluorescence. To examine the reproducibility of gyrase inhibition identified using the assay, we assembled a panel of 14 known gyrase inhibitors with IC_50_s spanning the range 1–200 µM (Table 1). Inhibition isotherms, based on the fluorescence of the reactions, were used to calculate an IC_50_ value for each inhibitor (Figure 5A, Table 1). In two independent replicate experiments, the MR of IC_50_s determined between runs for each inhibitor was 1.15; although this ratio is significantly different from 1.0 (95% confidence interval, 1.08–1.22), it represents a small difference in potency between runs and therefore demonstrates that there is good agreement between measurements in separate experiments (Figure 5B). For the cruciform extrusion assay, MSR = 1.30; a pair of compounds whose IC_50_ ratios are higher than this value can be judged to have different potency with 95% confidence. MSR values close to 1 indicate that an assay is useful for ranking compounds by potency; MSR ≤3 has been proposed as a practical limit for this criterion (32), a limit which our assay is well within. To compare potencies determined by the cruciform extrusion assay to those determined by the classic gel-based assay, IC_50_ values were also determined for ciprofloxacin and moxifloxacin using gel electrophoresis (Figure 6, Table 1). The resultant values proved to be in good agreement with those determined in the replicate-experiment study.

### Screening of the NIH Clinical Collection

**Figure 7.**
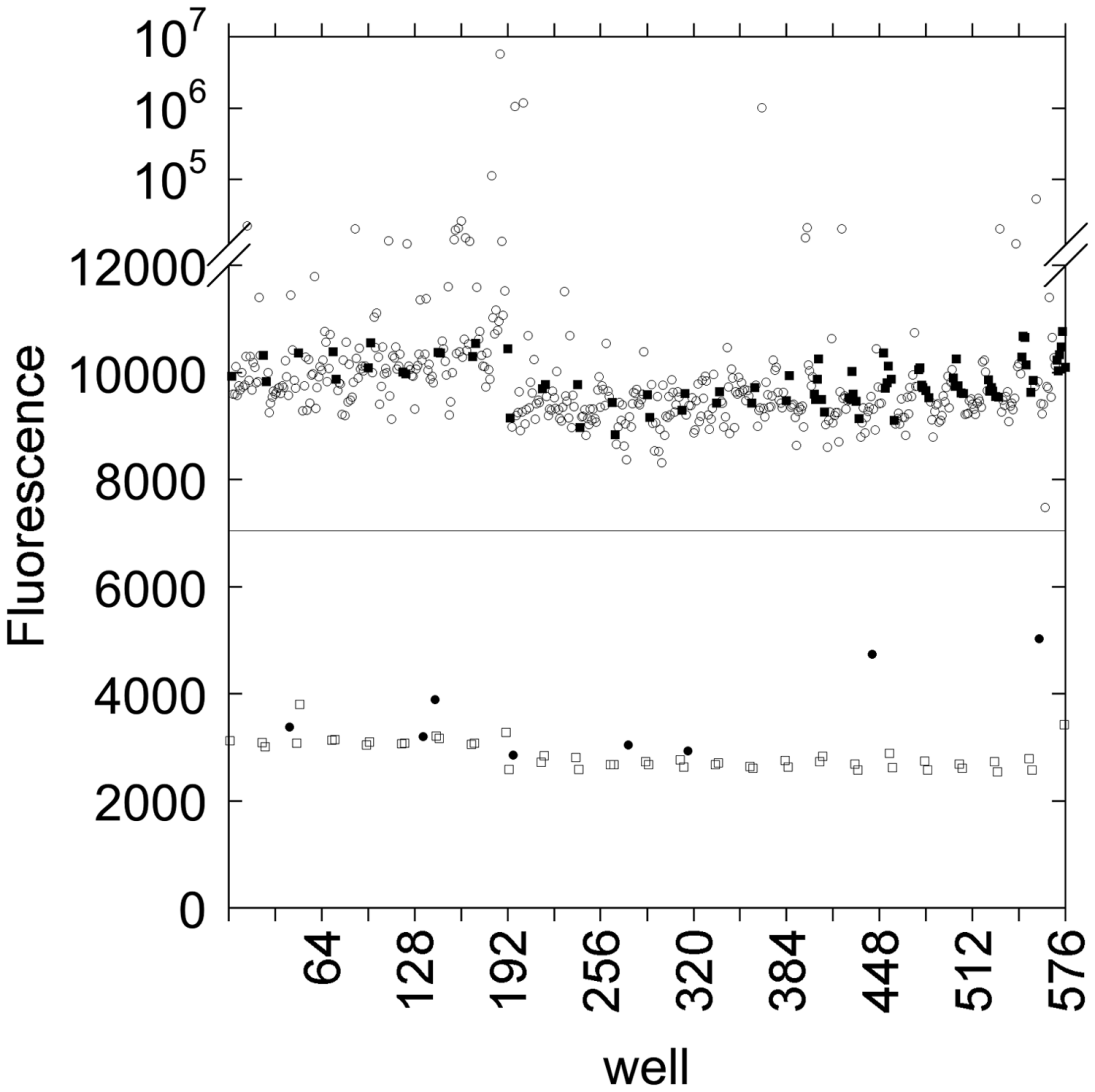
Results from screen of NCC. Plasmid pAT42C was incubated with DNA gyrase under supercoiling conditions in the presence of compounds from the NCC (circles); 1 mM ciprofloxacin was used as a positive inhibition control (open squares), and 1% (v/v) DMSO was included as a negative control (filled squares). The inherent fluorescence of many compounds in the NCC interfered with fluorescence from the supercoiled plasmid; in the future, this problem could be ameliorated by using a fluorophore with a red-shifted emission spectrum, e.g. TAMRA. Eight potential hits (filled circles) with >40% inhibition (solid line) were identified in this screen.

To assess the performance of the cruciform-extrusion HT assay against a broad range of compounds, the assay was next tested against the NIH Clinical Collection (NCC) using a drug concentration of 50 µM. The NCC contains 446 compounds, including seven fluoroquinolones that are known gyrase inhibitors. Ciprofloxacin (1 mM) was used as a positive control for 100% inhibition. The screen generated eight hits (Figure 7, Table 2), including the seven fluoroquinolones and cefixime. Cefixime is a cephalosporin; inhibition of gyrase by this drug was not reproducible in subsequent trials of the cruciform extrusion assay or the gel-based assay and was thus judged to be a spurious false positive.

**Table 2.** Positive results from screen of NCC

Compound	% Inhibition
Tosufloxacin	96%
Levofloxacin	99%
Rufloxacin	89%
Pazufloxacin	97%
Pefloxacin	95%
Moxifloxacin	96%
Cefixime	70%
Enrofloxacin	67%

### Calculation of Z’

The inherent quality of an assay is commonly represented by the Z’ factor (33). A Z’ > 0.5 is characteristic of an assay showing clear separation between positive and negative results. In six repetitions of the replicate-experiment study, Z’ ranged between 0.77 and 0.85. Similarly, in the three plates measured while screening the NCC, Z’ ranged from 0.816 to 0.839. To test the suitability of lower concentrations of ciprofloxacin as a positive control for gyrase inhibition, we repeated the control measurements of the NCC assay using 50 µM ciprofloxacin as a positive control and 1% DMSO as a negative control. When ciprofloxacin was used at the same concentration as the test compounds in the NCC, we calculated Z’ factors of 0.66, 0.81 and 0.84 for three replicate plates (Supplementary Figure S1). The clear separation between the positive and negative controls shows that the cruciform extrusion assay allows for the sensitive detection of supercoiling inhibitors.

## DISCUSSION

Although topoisomerases have long been the target of antimicrobial and antitumor agents, standard gel-based assays for monitoring DNA supercoiling and relaxation by these enzymes are both time-consuming and labor intensive. This shortcoming has led to the development of other assays that can read out on topoisomerase function—including fluorescent measurement of ethidium bromide binding (23), induction of SOS response (34) and fluorescence anisotropy measurement of DNA triplex formation (35,36)—for HT screening of potential inhibitors of these enzymes.

We describe here the development of a new HT assay for DNA supercoiling based on the extrusion of a cruciform from a plasmid. Labeling of the cruciform with a fluorophore and quencher at specific positions results in an increase in fluorescent signal on extrusion of the cruciform that accompanies supercoiling of the plasmid. This event in turn allows real-time monitoring of plasmid supercoiling by gyrase (Figure 1). One advantage of our cruciform extrusion assay compared with existing HT supercoiling assays is the high Z’ score, which is due both to a ∼4-fold range of signal between the positive and negative controls and to the low standard deviations of these measurements. A high Z’ score indicates a sensitive HT assay, reflecting the quality of the positive and negative controls and the reproducibility of the data points.

An additional utility of the cruciform extrusion assay as a primary screening tool is the use of fluorescence quenching as an indication of inhibition. Because many potential inhibitors are themselves fluorescent, a property we observed in our assessment of the NCC (Figure 7), an assay that relies on either gain of fluorescence or loss of fluorescence anisotropy to identify inhibitors stands to identify a large number of false-positive hits. Although it is possible that some naturally fluorescent inhibitors may be incorrectly ruled out, the likelihood of this occurrence could be reduced by using a longer wavelength fluorophore–quencher pair than we have used here, such as TAMRA and Black Hole Quencher 1 (37). Molecules that quench fluorescence, absorb light or alter DNA topology may appear as false-positive hits in the cruciform extrusion assay. Shapiro and colleagues (38) have described a method to correct for such artifacts by measuring the signal in an artifact plate. In our application, an artifact plate would be identical to a test plate, except that enzyme would be omitted and supercoiled pAT42C (product) would be substituted for relaxed pAT42C (substrate).

Although the high Z’ score and real-time capabilities of the cruciform extrusion assay represent an advance over existing HT assays, there are limitations as well. Unlike the ethidium bromide intercalation assay (23), the cruciform extrusion assay as implemented in this work cannot discriminate between catalytic inhibitors and gyrase poisons that cleave DNA; both types of agent result in a quenched product. Additionally, unlike the ethidium bromide intercalation and triplex formation (24) assays, the cruciform extrusion assay is binary: it can discriminate between DNAs with σ < −0.04 and σ > −0.04, but it cannot report with precision on the supercoiling density and/or distribution within a population of DNA, nor can it detect changes in the supercoiling density of positively supercoiled DNA.

We envision that our strategy of constructing fluorescently labeled cruciforms could have utility beyond in HT assays for gyrase inhibitors. By changing the relative positions of the fluorophore–quencher pair and tuning the degree of negative supercoiling of the plasmid, we could enable real-time detection of other processes that result in a change in the writhe of DNA. Such processes may include supercoil relaxation by other type I and type II topoisomerases, nucleosome assembly (39,40) and DNA remodeling (41,42).

In summary, we have demonstrated that cruciform extrusion can be used as a near-real time reporter of topological transitions of closed-circular DNA. By using a cruciform-containing plasmid labeled with a fluorophore and quencher, we have observed fluorescence changes corresponding to the introduction or removal of negative supercoils by *E.coli* gyrase and human topo IIα, respectively. We have subsequently implemented this substrate in a HT assay for DNA supercoiling by gyrase. The HT assay reported here may be applied to the search for novel antibacterial agents that inhibit gyrase and may be extendable to the search for antitumor inhibitors of human topo II and topo I.

## SUPPLEMENTARY DATA

Supplementary Data are available at NAR Online: Supplementary Figure 1.

## FUNDING

National Institutes of Health (NIH) [5R01AI091412 to J.M.B.]. Funding for open access charge: NIH.

*Conflict of interest statement*. K.M.J. and J.M.B. are co-founders of Replisoma, Inc.

## Supplementary Material

Supplementary Data
