# Supplementary material for: Real-time detection of DNA topological changes with a fluorescently labeled cruciform
Source: Nucleic Acids Res. 2013 May 16;41(13):e133. doi: 10.1093/nar/gkt413 (PMC3711437; doi:10.1093/nar/gkt413)
Supplement: Supplementary Data [file supp_41_13_e133__index.html]

Real-time detection of DNA topological changes with a fluorescently labeled cruciform — Real-time detection of DNA topological changes with a fluorescently labeled cruciform — Supplementary Data 

# Real-time detection of DNA topological changes with a fluorescently labeled cruciform

## Supplementary Data

files

**Files in this Data Supplement:**

- Supplementary Data - pdf file
