# Supplementary material for: Real-time detection of DNA topological changes with a fluorescently labeled cruciform
Source: Nucleic Acids Res. 2013 May 16;41(13):e133. doi: 10.1093/nar/gkt413 (PMC3711437; doi:10.1093/nar/gkt413)

Supplementary Figure S1. Plasmid pAT42C was incubated with DNA gyrase under conditions identical to those in Figure 7. 50 μM ciprofloxacin was used as a positive inhibition control (open squares), and 1% DMSO was used as a negative control (filled squares). Mean values for positive and negative controls from three replicates are indicated by solid lines.

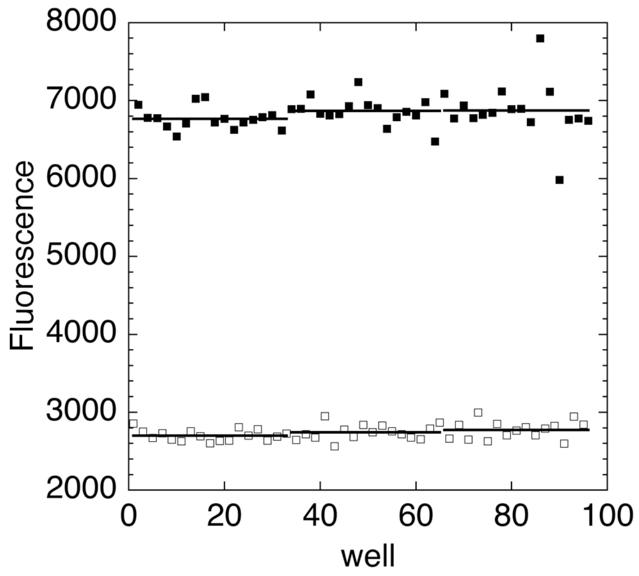

Supplement: Supplementary Data [file supp_gkt413_nar-00427-met-k-2013-File009.pdf]
